# Supplementary material for: Polymorphisms in Pfkelch13 domains before and after the introduction of artemisinin-based combination therapy in Southwest Nigeria
Source: PLoS One. 2025 Mar 31;20(3):e0316479. doi: 10.1371/journal.pone.0316479 (PMC11957316; doi:10.1371/journal.pone.0316479)

Samples: 14074
Bases: 826
Average spacing: 18

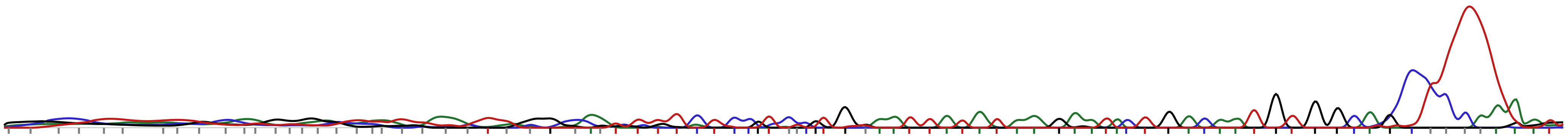

10 20 30 40 50 60 70 80

- T - - G - A - T T T T C T C C G T - - C G T G - A A T T A T A T A A G A A T A C T G A C A A T G T G G C A G C - - - - A A A T

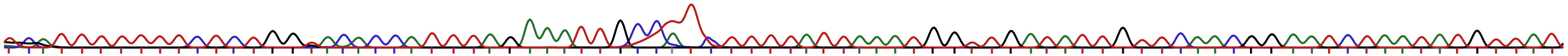

90 100 110 120 130 140 150 160 170

T C A T T T T T T T C T C T G G T A C A C C A T T T A G A A A T T G C C - T C T T T T A T T A A A T G G T T G A T A T T G T T C A A C G G A A T C T A A T A T G T T A T

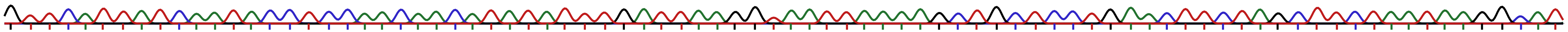

180 190 200 210 220 230 240 250

G T T C A T T A T C A A T A C C T C C A A C A A C A T A T A T T T G A T T A A G G T A A T T A A A A G C T G C T C C T G A A C T T C T A G C T T C T A A T A A G G C A T

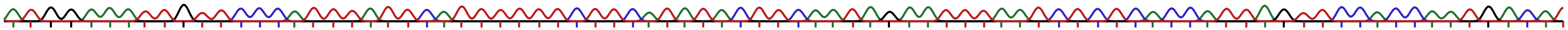

260 270 280 290 300 310 320 330

A T G G A A A T T G T T C C C A T T T A T T C A T T T T T T C T T C A T A T A C T T C A A T A G A A T T T A A T C T C T C A C C A T T A G T T C C A C C A A T G A C A T

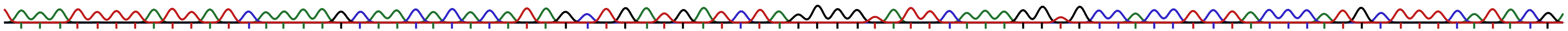

340 350 360 370 380 390 400 410 420

A A A T T T T A T T A T C A A A A G C A A C A C A C A T A G C T G A T G A T C T A G G G G T A T T C A A A G G T G C C A C C T C T A C C C A T G C T T T C A T A C G

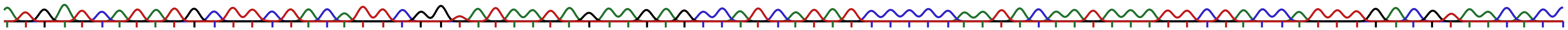

430 440 450 460 470 480 490 500

A T G A T C A T A T G C T T C T A C A T T C G G T A T A A T A G A A G A G C C A T C A T A T C C C C A A T A C A A T A A A T T C T A C C A T T T G A C G T A A C A C C

Samples: 14074
Bases: 826
Average spacing: 18

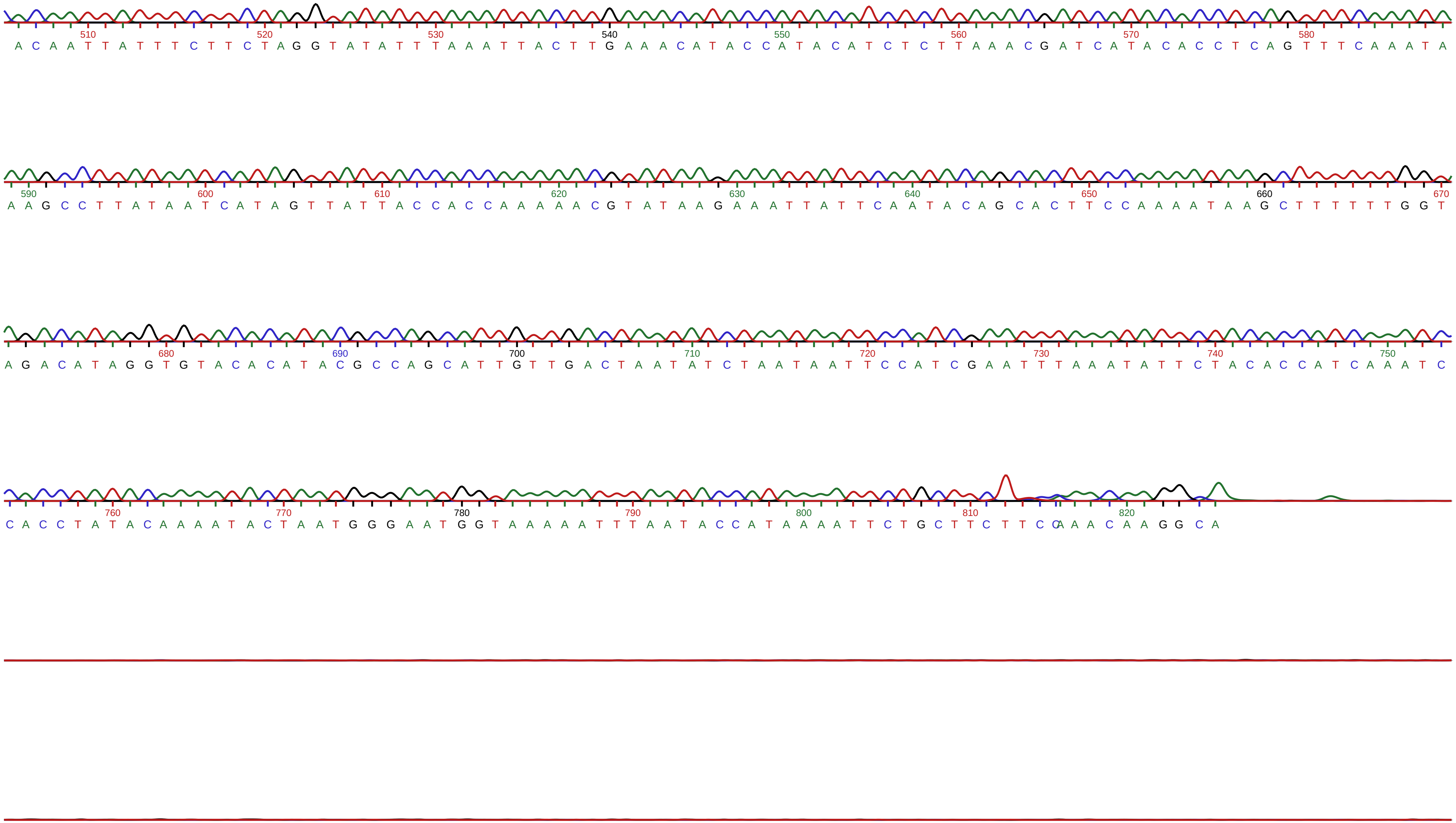

Samples: 14074
Bases: 826
Average spacing: 18

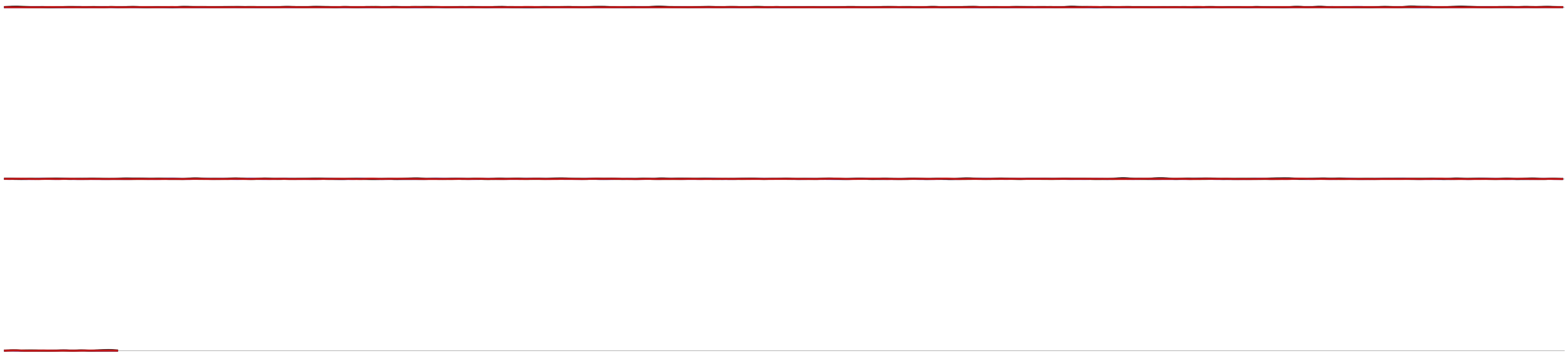

Supplement: Supporting information 2 — (ZIP) [file pone.0316479.s002.zip › 035KN1R_PREMIX_Plate_KELCH1_C04.pdf]
